# Photocatalytic and antibacterial activity of graphene oxide/cellulose-doped TiO_2_ quantum dots: *in silico* molecular docking studies†

**DOI:** 10.1039/d2na00383j

**Published:** 2022-07-27

**Authors:** Muhammad Ikram, Fahad Rasheed, Ali Haider, Sadia Naz, Anwar Ul-Hamid, Anum Shahzadi, Junaid Haider, Iram Shahzadi, Shaukat Hayat, Salamat Ali

**Affiliations:** Solar Cell Applications Research Lab, Department of Physics, Government College University Lahore Lahore 54000 Punjab Pakistan dr.muhammadikram@gcu.edu.pk; Department of Physics, Riphah Institute of Computing and Applied Sciences (RICAS), Riphah International University 14 Ali Road Lahore Pakistan; Department of Clinical Sciences, Faculty of Veterinary and Animal Sciences, Muhammad Nawaz Shareef University of Agriculture Multan 6000 Pakistan; Tianjin Institute of Industrial Biotechnology, Chinese Academy of Sciences Tianjin 300308 China; Core Research Facilities, King Fahd University of Petroleum & Minerals Dhahran 31261 Saudi Arabia anwar@kfupm.edu.sa; Faculty of Pharmacy, The University of Lahore Lahore Pakistan; Punjab University College of Pharmacy, University of the Punjab 54000 Pakistan

## Abstract

Graphene oxide (GO) and cellulose nanocrystal (CNC)-doped TiO_2_ quantum dots (QDs) were effectively synthesized by employing the co-precipitation method for the degradation of dyes and antimicrobial applications. A series of characterizations, *i.e.*, XRD, FTIR, UV-visible spectroscopy, EDS, FE-SEM, and HR-TEM, was used to characterize the prepared samples. A reduction in PL intensity was observed, while the band gap energy (*E*_g_) decreased from 3.22 to 2.96 eV upon the incorporation of GO/CNC in TiO_2_. In the Raman spectra, the D and G bands were detected, indicating the presence of graphene oxide in the composites. Upon doping, the crystallinity of TiO_2_ increased. HR-TEM was employed to estimate the interlayer *d*-spacing of the nanocomposites, which matched well with the XRD data. The photocatalytic potential of the prepared samples was tested against methylene blue, methylene violet, and ciprofloxacin (MB:MV:CF) when exposed to visible light for a certain period. The antibacterial activity of GO/CNC/TiO_2_ QDs against *Staphylococcus aureus* (*S. aureus*) and *Escherichia coli* (*E. coli*) bacteria *in vitro* was tested to determine their potential for medicinal applications. The molecular docking investigations of CNC-TiO_2_ and GO/CNC-doped TiO_2_ against DNA gyrase and FabI from *E. coli* and *S. aureus* were found to be consistent with the results of the *in vitro* bactericidal activity test. We believe that the prepared nanocomposites will be highly efficient for wastewater treatment and antimicrobial activities.

## Abstract 2

Graphene oxide (GO) and cellulose nanocrystal (CNC)-doped TiO_2_ quantum dots (QDs) were effectively synthesized by employing the co-precipitation method for the degradation of dyes and antimicrobial applications.

## Graphical abstract

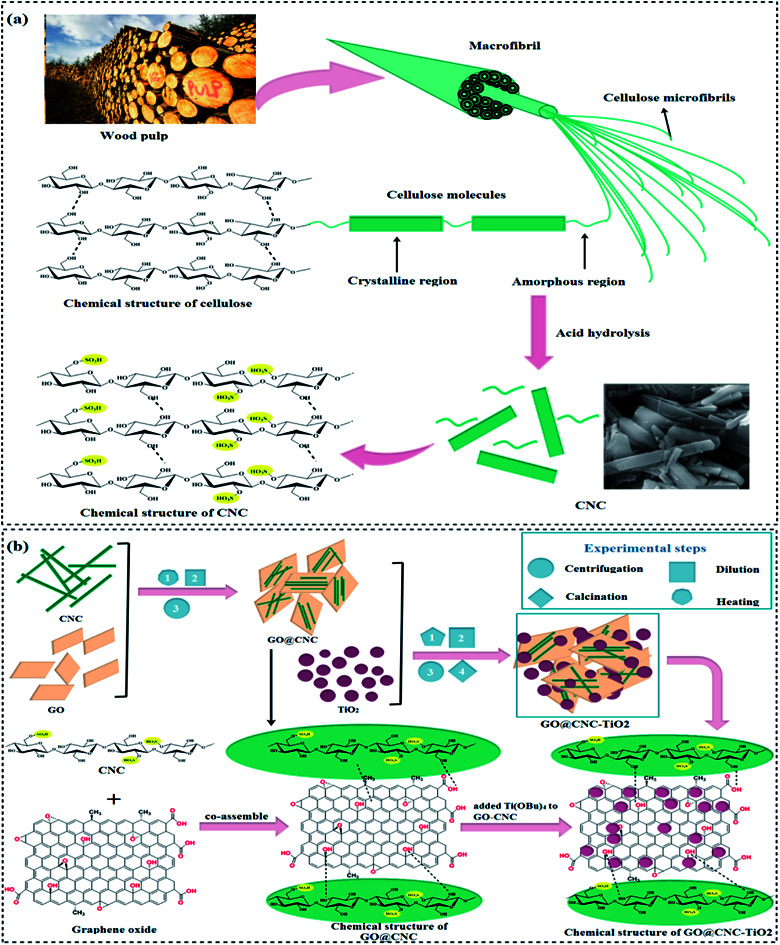

## 1. Introduction

One of the most effective and viable ways to control water treatment is through catalytic treatment using light-induced oxidation to purify water. The use of nanomaterials, mainly semiconductors such as TiO_2_, ZnS, CdS, and CeO_2_ as potential photocatalysts, has attracted significant attention over the last few decades.^1^ Among them, TiO_2_ is the preferred material for application in photovoltaics, water splitting, and treatment of aqueous pollutants because of its cost-benefit ratio, high oxidizing capability, and long-term corrosion resistance.

Generally, the factors affecting the photocatalytic activity of TiO_2_ include its energy band gap, photogenerated excitons, lifetime, and large surface area.^1–3^ Large band gap values (3.0 and 3.2 eV) and reduced quantum efficiency impair the photocatalytic performance of rutile and anatase TiO_2_ structures.^4^ However, achieving a large band gap is a significant challenge. Accordingly, doping and dye-sensitization are the most common ways to enhance its optical response in the visible light region. In general, energy intensive methods are utilized for doping other elements in TiO_2_, such as hydrothermal treatment and high-temperature annealing. Dye-sensitization necessitates that dye molecules, when irradiated, transport photoexcited electrons to the conduction band (CB) of TiO_2_. Furthermore, the dye molecules in water environment must be very stable. However, upon dye sensitization, charge transfer in visible light-induced photocatalysis occurs from the surface of the complex rather than the absorption of visible light through self-sensing. Consequently, many chemicals not absorb visible light, making them plausible sensitization candidates.^5–8^

Different approaches have been suggested in the last decade to overcome the above-mentioned disadvantage of TiO_2_ including doping with non-metals and metals, sensitizing through dyes and compositing with materials such as carbon nanotubes (CNTs) and graphene. Among them, due to the high specific surface area and electrical conductivity of graphene, when it is doped in TiO_2_, it accepts and transmits photo-generated electrons from TiO_2_, enhancing the charge separation.^9–12^ In this case, the structure of TiO_2_ would considerably improve the photocatalytic activity under visible light in the presence of another element, therefore adjusting its higher *E*_g_ (UV area) to a lower *E*_g_ (visible light region).

Another material used for this investigation is cellulose nanocrystal (CNC), which possesses exceptional mechanical strength, a wide surface area, and negatively charged sulfuric groups (besides hydroxyl groups being present on the surface of CNC).^5^ Because of its enhanced crystallinity and good interfacial interaction, CNC exhibits potential to significantly increase the mechanical performance, thermal stability, barrier characteristics, and optical strength of composites. Various studies confirmed that the homogeneous mixing of CNC and GO results in their good integration through hydrogen bonding and intermolecular interactions, and the presence of abundant oxygen-containing groups in GO-CNC composites make them very hydrophilic and allow them to be easily dispersed in water and other organic solvents.^13–15^ Similarly, Lv *et al.* concluded that the hydrogen bonding interaction between the hydroxyl groups of CNC and GO sheets facilitates their excellent hydrophilicity by the efficient dispersion of GO sheets and also improves the negative zeta-potential of the resulting composite.^16^ CNC can also be exploited to stabilize nanoparticles with unique functionality for specific applications. Furthermore, CNC has recently been explored for application in drug delivery systems due to its biocompatibility, biodegradability, and absence of cytotoxicity.^17,18^

It is well established that most bacterial cell walls (*E. coli* and *S. aureus*) are negatively charged. Indeed, certain substances (quaternary ammonium compounds, molecules, and polymers) can electrostatically interface with the negatively charged bacterial surface, and therefore induce membrane rupture and eventual posterior death. The presence of hydroxyl groups makes it ideal for active ingredients and water molecules to connect in a hydrogen-bonded network, encouraging these chemicals to bind, and subsequently transfer to the skin.^19^ Recently, nanoparticles (NPs) have been employed as an alternative strategy for the treatment of infections caused by resistant microorganisms owing to their unique physical and chemical characteristics. Considering the therapeutic potential of NPs and nanocomposite systems, which promise potentially wide applications in biomedical sciences, a clear understanding of their mechanism of action is desirable. Given that enzymes represent key virulence factors responsible for various microbial infections, inhibiting their activity will aid in finding treatments for diseases. NPs tend to inhibit the activity of various enzymes essential for bacterial growth by binding inside their active pocket.^20,21^ Accordingly, targeting enzymes belonging to key biosynthetic pathways represents an effective strategy to identify the effects of these NPs on their activity and to explore the potential of these NPs as possible inhibitors and antibacterial agents. The use of computational techniques such as molecular docking studies for a clear understanding of the underlying mechanism of the bactericidal activity of nanocomposite systems is well documented.^22,23^ Based on this background, we believe that use of GO/CNC co-doped TiO_2_ QDs as a photocatalyst in combination with ultrasound irradiation to treat wastewater is promising. Further, to the best of our knowledge, the fabrication of GO/CNC-doped TiO_2_ QDs and their antimicrobial activity and use for the degradation of organic contaminants have not been reported to date.

This study aimed to co-precipitate GO/CNC-doped TiO_2_ nanostructures and test their photocatalytic and antibacterial properties. *In silico* molecular docking experiments were performed against specific enzyme targets from the fatty acid and nucleic acid biosynthesis pathways to uncover their probable bactericidal mechanisms.

## 2. Experimental

### 2.1. Materials

Cellulose nanocrystals (CNC, C_6_H_10_O_5_, size of ∼50 μm), titanium(iv) *n*-butoxide (Ti(OBu))_4_, and sodium hydroxide pellets (NaOH) were obtained from Sigma Aldrich. GO was synthesized in the laboratory. Sulfuric acid (H_2_SO_4_) and hydrochloric acid (HCl) 99.9% and 37%, respectively, were obtained from Analar (USA). All reagents and chemicals were laboratory grade, and thus utilized as received without further purification.

### 2.2. Synthesis of cellulose nanostructured crystals (CNC)

**Fig. 1:**
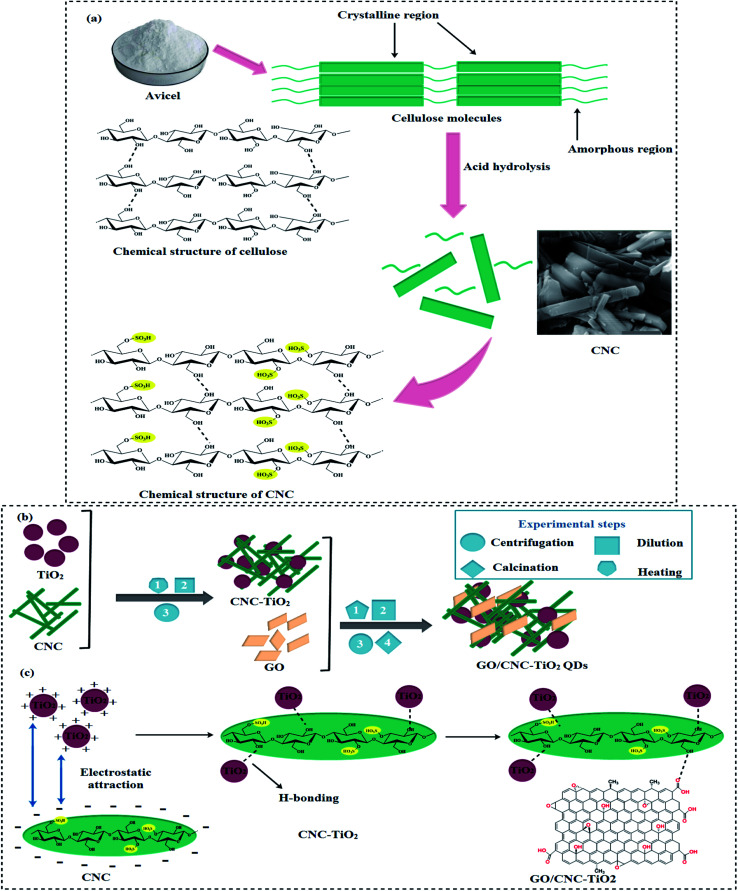
(a) Synthesis of cellulose nanocrystals, (b) schematic diagram of GO/CNC–TiO_2_ synthesis and (c) chemical structure of synthesized GO/CNC–TiO_2_ QDs.

Avicel (10 g) was hydrolyzed with 50% H_2_SO_4_ solution under heat treatment at 70 °C for 30 min to produce CNC, together with the hydrolysis technique. During hydrolysis, a colloidal solution was poured into 5000 mL of deionized water, then centrifuged (7100 rpm, 10 min), and the supernatant was poured off. Centrifugation was repeated to achieve neutral pH in the suspension through NaOH (Fig. 1a). Finally, a CNC suspension (dispersing 98 mg mL^−1^) was obtained.^24^

### 2.3. Synthesis of GO/CNC-doped TiO_2_ QDs

The typical procedure for the synthesis of GO/CNC-doped TiO_2_ is as follows: GO sheets were dispersed by ultrasonication for 30 min.^25^ Two gradual centrifugations were performed to separate the large graphene oxide sheets with a size of 3–5 μm. Firstly, centrifugation was performed at 600 rpm for 3 min to remove the unreacted sediments, and then at 3000 rpm for 3 min to recover the large sheets with a thickness of ∼1 nm, as shown by atomic force microscopy (AFM) in Fig. S1.† After the addition of CNC with a concentration of 2.55 mol L^−1^, the CNC suspension was poured in the desired amounts of Ti(OBu)_4_ and C_2_H_5_OH (50 mL), while HCl (0.8 mL) was used to control the pH. Subsequently, GO sheets were added to the CNC-doped TiO_2_ solution. This mixture was kept under vigorous stirring at 70 °C for 30 min, and a pre-prepared 0.1 M solution of NaOH was used to maintain the pH value at 7. After centrifuging and washing with DI water, the product was dried to obtain GO/CNC-TiO_2_ quantum dots (QDs).^24,26^ Finally, the samples were calcined at 400 °C for 4 h and ground to a fine powder, as shown in Fig. 1b. The reaction between CNC and TiO_2_ occurred in two stages: firstly, particles were attracted to one another by long-range electrostatic forces, and afterward hydrogen bonds were formed. GO was bonded to the CNC-TiO_2_ composites *via* hydrogen bonding to generate GO/CNC–TiO_2_ QDs (Fig. 1c).

### 2.4. Photocatalytic activity

The photocatalytic potential of the doped QDs was tested against a combination of methylene blue (MB) and methylene violet (MV) organic dyes and ciprofloxacin (CF) as an antibiotic under light illumination. MB, MV, and CF were prepared by dissolving 3 mg each in DI water (1000 mL) in the dark. A small amount (3 mL) of the prepared sample was taken as a reference sample for a comparative study. Subsequently, 10 mg of prepared sample was added to the dye (40 mL) and the sample mixed dye solution was fixed under a mercury lamp (400 W), where the distance between the sample and lamp was ∼20 cm to observe the dye degradation. The change in the blue color of the dye solution to colorless indicated the degradation of the dye. After exposure to illumination, 3 mL of dye was drawn from the solution and examined under a UV-Vis spectrophotometer at regular intervals. The reduction in *λ*_max_ = 665 nm of the dye solution indicated the effective dye degradation in the presence of the nanocatalysts.

### 2.5. Isolation and identification of *S. aureus* and *E. coli*

Caprine milk specimens that were clearly positive for mastitis after confirmation by the Surf Field Mastitis Test (SFMT) were acquired from local farms in Punjab, Pakistan. Initially, the specimen was cultured on 5% blood agar, and later on specific media (MacConkey agar (MA) and mannitol salt agar (MSA)) for segregation of the refined *S. aureus* and *E. coli* in triplets. Taxonomical and biochemical identification were carried out with Gram staining, catalase, and coagulase tests.^26^

### 2.6. Antimicrobial activity

The well diffusion procedure was adopted after swabbing 0.5 McFarland (1.5 × 10^8^ CFU mL^−1^) of discrete Gram −ve and +ve isolates on MA and MSA plates to evaluate the *in vitro* bactericidal activity of the GO/CNC-doped TiO_2_ QDs. Specific GO/CNC-doped TiO_2_ (50 μL) concentrations of 1% and 2% were loaded as the minimum and maximum dosages on the sterilized MA and MSA plates having 6 mm wells made with sterilized well borer in comparison with DIW and ciprofloxacin (0.33%) as the −ve and +ve controls, respectively. The bactericidal potency was validated by assessing the inhibition areas (mm) after incubation in dishes for 12 h at 37 °C utilizing the Vernier scale. The microcidal effectiveness of the GO/CNC-doped TiO_2_ was measured as inhibition area (mm) and examined statistically with one-way variance analysis (ANOVA) considering *p* < 0.05.

### 2.7. *In silico* analyses to investigate antimicrobial activity

Enzymes belonging to the nucleic acid and fatty acid biosynthesis pathways, as attractive targets for the discovery of antibiotics,^27,28^ were selected for molecular docking predictions of the TiO_2_-CNC and GO/CNC-doped TiO_2_ nanocomposites. Molecular docking was performed using the MOE software.^26,29^ The 3D-structural coordinates of the enzyme targets were obtained from https://www.rcsb.org/ with PDB ID as 5MMN for DNA gyrase,^30^1MFP^31^ and 6TBC^32^ for enoyl-[acyl carrier-protein] reductase (FabI) from *E. coli* and *S. aureus*, respectively. The preparation of the enzyme structures was done using the energy minimization tool of MOE, whereas the default algorithm (gradient: 0.05, force field: MMFF94X) was employed for 3D-protonation. The binding pocket was specified around native ligand, *i.e.*, 1-ethyl-3-[8-methyl-5-(2-methyl-pyridin-4-yl)-isoquinolin-3-yl]-urea for DNA gyrase, indole naphthyridinone (IDN) and kalimantacin B for FabI of *E. coli* and *S. aureus*, respectively. Discovery Studio and MOE Visualizer together with pymol were used for the analysis of the docked complexes and 3D-view representation of the binding patterns of the nanocomposites with active site residues.^33^ The builder tool of MOE was employed for the structure preparation of the nanocomposite ligands.

## 3. Results and discussion

**Fig. 2:**
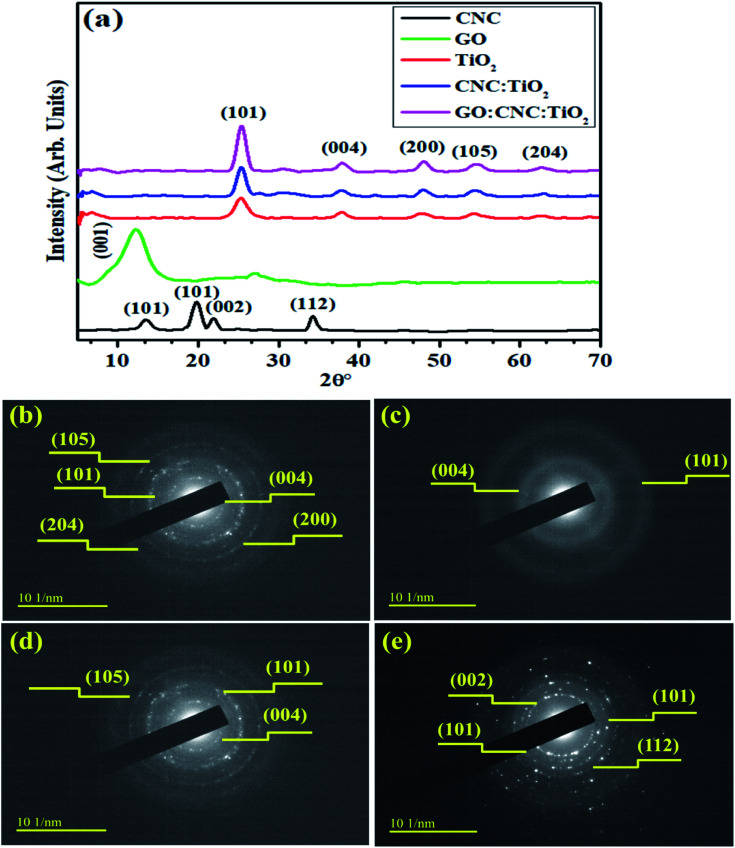
(a) XRD patterns and (b–e) SAED patterns of CNC, TiO_2_ CNC/TiO_2_ and GO/CNC-doped TiO_2_ QDs, respectively.

**Fig. 3:**
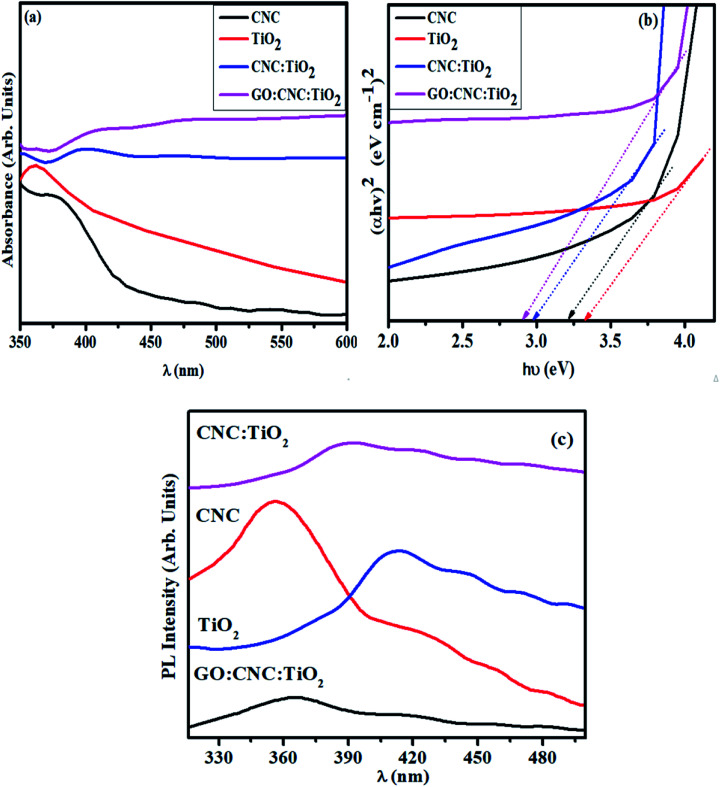
(a) Absorption spectra of undoped and GO/CNC-doped TiO_2_ QDs, (b) band gap plot and (c) PL spectra.

XRD was employed to examine the crystalline nature, phase constitution and crystallite size of the samples in the 2*θ* range of (5–70°), as shown in Fig. 2a. The peaks located at 12° (101), 19.5° (101), 22.4° (002) and 33.9° (112) are ascribed to the cellulose nanocrystals (CNC), as indicated in JCPDS Card No.: 86-1157.^34^ In the XRD analysis, the diffraction peak of GO was observed ∼2*θ* = 12.33° (Fig. 3a), which corresponds to the (001) plane, indicating the presence of the hexagonal phase, which is consistent with JCPDS card # 04-0783.^35^ The diffraction peaks of TiO_2_ observed at 25.47°, 37.79°, 48.18°, 53.87°, and 62.68° are indexed as the (101), (004), (200), (105), and (204) planes, respectively, confirming the tetragonal structure of anatase TiO_2_ (JCPDS card 21-1272).^36^ Improved crystallinity was observed upon the doping of GO/CNC in the pristine material. The diffraction peaks of CNC and GO were not observed in the doped samples because of their low doping amount, but good dispersion and increased diffraction intensity of CNC:TiO_2_ (101) were detected.^37^ The crystallite size of the samples was evaluated using the Debye–Scherer formula. The crystallite size of CNC, TiO_2_, CNC/TiO_2_, and GO/CNC-doped TiO_2_ was estimated to be 10.11, 4.36, 5.97, and 6.28 nm, respectively, which increased gradually with doping. The selected area electron diffraction (SAED) patterns of the doped and pristine TiO_2_ QDs with concentric rings were attributed to the different planes of the XRD patterns (Fig. 2b–e). These circular rings with bright spots demonstrated the polycrystalline nature of CNC, TiO_2_, and CNC/TiO_2_. With the incorporation of GO, the crystallinity of the concerned samples was enhanced, as revealed by the XRD data.

The absorption spectra of the synthesized samples measured using a UV-Vis spectrophotometer are shown in Fig. 3a. The absorption peaks of CNC and TiO_2_ were found at around 370 and 380 nm, respectively.^34,36^ The peaks between 360–420 nm in the GO/CNC-doped TiO_2_ are attributed to the π → π* transition of the atomic C–C ring and C–O bond. Moreover, the lone pairs of electrons enabling n → π* transitions resulted in a further redshift in the peaks (360–460 nm) in CNC:TiO_2_.^38^ The absorption range increased in the visible range upon doping GO in CNC–TiO_2_, showing improved light-harvesting ability. This can be attributed to the relatively easy excitation of the lone electrons in contrast to the bonded electrons, resulting in more n → π* as compared to π → π* transitions.^25^ A decrease in bandgap energy (*E*_g_) from 3.2 to 2.96 eV was observed for the doped samples,^34,36,39^ which can be attributed to the quantum confinement effect and increase in particle size upon GO/CNC doping in the control sample (Fig. 3b).^40^ Furthermore, efficiency of electron–hole pair recombination was characterized through photoluminescence spectrum, where a lower peak intensity designates a lower exciton recombination rate and effective dissociation of charge carriers, leading to an efficient photocatalytic performance. At the excitation wavelength of *λ*_max_ = 300 nm, various emission peaks were observed, as shown in Fig. 3c. The extremely small size of the synthesized QDs compliment the quantum confinement effect, which is prominent in the PL emission spectra, given that energy level quantization and band gap reduction are major consequences of confinement in QDs.^41^ The PL emission spectrum of CNC with peaks positioned near 353, 410, and 465 nm and its contributions to the total spectrum are highly dependent on *λ*_ex_, which can be attributed to the carbonyl groups and various low-molecular derivatives of CNC.^42^ The PL spectra with peaks at 341, 400, 440, 467, and 488 nm for TiO_2_ and the doped samples are ascribed to oxygen vacancies and excitons (self-trapped) localized on TiO_6_ octahedra.^43^ The defect PL intensity was quenched when the Ti^+^ ions were irradiated with light due to the disorder and turbulence in the lattice, agreeing well with the Raman spectra of the GO/CNC:TiO_2_ QDs. The peak intensity of TiO_2_ was higher compared to that of CNC:TiO_2_, suggesting that exciton recombination was sharply reduced and electrons with a longer life were generated. The GO/CNC:TiO_2_ sample exhibited the lowest PL intensity, indicating the highest charge carrier separation efficiency and superior photocatalytic activity.^44^

**Fig. 4:**
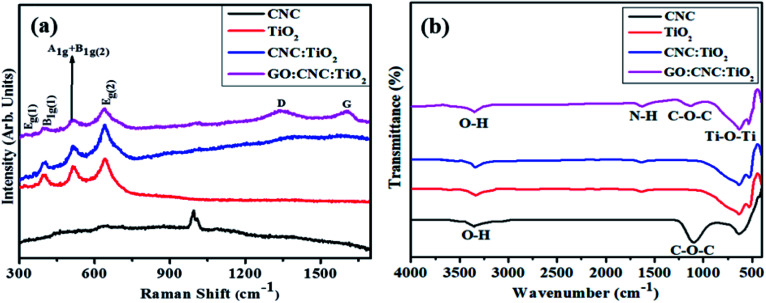
(a) Raman spectra and (b) FTIR analysis of pristine CNC, TiO_2_, CNC@TiO_2_, and GO/CNC co-doped TiO_2_.

Additionally, the structural and chemical properties were determined by analyzing the Raman spectra of all the prepared samples (Fig. 4a). A strong Raman band centered at ∼1005 cm^−1^ and numerous weak bands were observed at ∼200 to 670 cm^−1^, confirming the presence of CNC in the mapped area. The band observed at ∼1005 cm^−1^ is ascribed to the β-1,4 glycosidic linkage (C–O–C) stretching modes and C–O ring stretching modes of the glucose rings present in the cellulose chains.^45^ Furthermore, the bands detected at ∼200–670 cm^−1^ are ascribed to the skeletal bending ((CCC, COC, and OCC)) and stretching (CC and CO) modes, confirming the formation of CNC.^46^ E_g(1)_, B_1g(1)_, A_1(g)_ + B_1g(2)_, and E_g(2)_ are the Raman vibrational mode matching symmetries, corresponding to the crystalline anatase TiO_2_ phase.^47^ Upon the doping of GO/CNC, a decrease in band intensity was also observed. The peaks detected at 1585 cm^−1^ (G band) and 1345 cm^−1^ (D band) also indicate the existence of the GO/CNC composites. The D band suggests the presence of sp^3^ defects in carbon atoms and the G peak gives information about the vibrations of the in-plane C=C (sp^2^ hybridized) bonds of carbon-based materials.^48^ An enhancement in the G and D bands was observed for the doped samples. Moreover, the high *I*_D_/*I*_G_ ratio was attributed to the increased number of sp^2^ domains.^49^

The FTIR spectra exhibited the attached functional groups, adsorbed molecules and impurities on surface-activated bonding, consistent with the molecular vibration modes (Fig. 4b). The transmission bands of CNC were observed at 3400 and 1110 cm^−1^, representing the aromatic bands. The characteristic peak appearing at 3340 cm^−1^ is ascribed to the O–H (acid) stretching bonding due to the hydrogen bonding hydroxyl group vibrations. The transmission peak found at 1110 cm^−1^ was obtained from the stretching vibration of the C–O–C pyranose ring.^50^ The broad band of TiO_2_ observed at 3432 and 1629 cm^−1^ indicated O–H and N–H groups, respectively, adsorbed on the sample surface. The presence of a strong band in the range of 1000–450 cm^−1^ revealed that the Ti–O–Ti stretching modes shifted towards lower wavenumbers as a result of the combined effects of the Ti–O–Ti and Ti–O–C vibrations, indicating that GO/CNC was successfully doped. This band shift confirms the chemical reaction between TiO_2_ (surface hydroxyl groups) and GO/CNC (functional groups).^36,51^

**Fig. 5:**
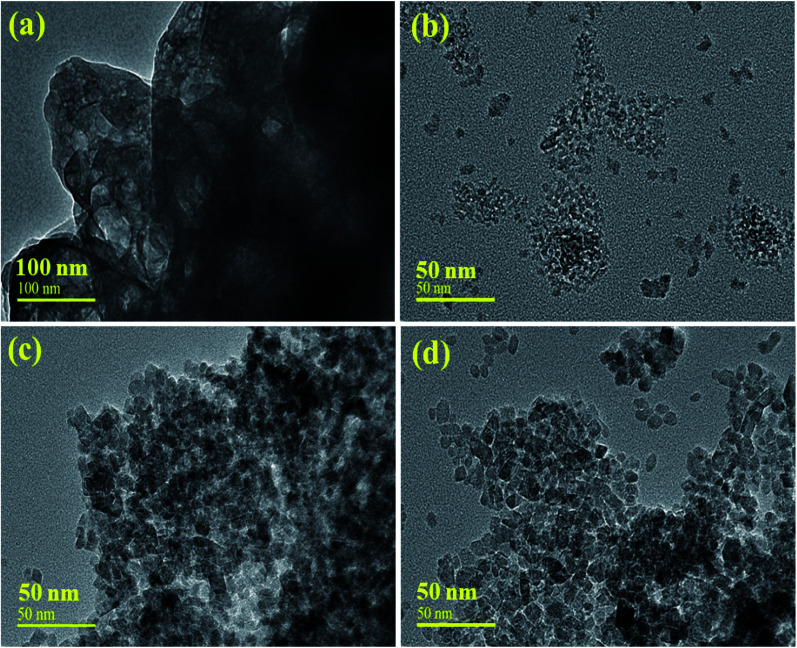
(a) HR-TEM images of CNC, (b) TiO_2_, (c) CNC@TiO_2_ and (d) GO/CNC-doped TiO_2_ QDs.

The morphology of the prepared samples was revealed through HR-TEM, as depicted in Fig. 5(a and b). The obtained images of CNC and TiO_2_ display the structure of the particles with higher agglomerations (Fig. 5a), while the HRTEM image of TiO_2_ shows mostly the homogeneous size of QDs with a few chunks present, as shown in Fig. 5b. It was observed that the inclusion of CNC and GO increased the diameter of the spherical QDs, which were surrounded by CNC and GO sheets Fig. 5(c and d), respectively. The material may have an excessive number of active sites for reactants, which can considerably increase its catalytic potential.^52^ Further, the size of TiO_2_ and the CNC QDs was examined using imaging software, which computed the estimated values of 1.52 to 10.04 nm and 1.02 to 3.17 nm in length, respectively.

**Fig. 6:**
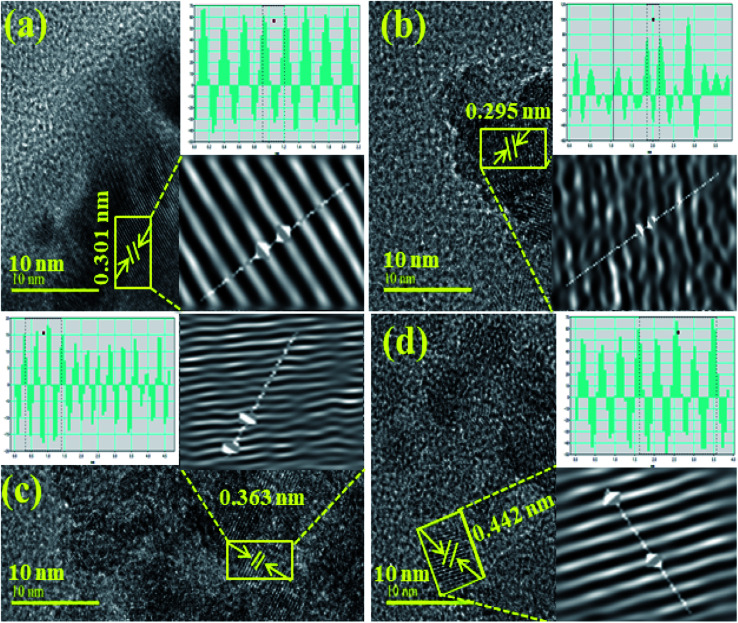
Calculated d-spacings using HR-TEM images of (a) CNC, (b) TiO_2_, (c) CNC:TiO_2_ and (d) GO/CNC:TiO_2_.

The interlayer *d*-spacing was calculated using the Gatan Digital-Micrograph software using the HR-TEM images, as shown in Fig. 6a–d. The d-spacing value for the pristine TiO_2_ was 0.295 nm, which agreed well with the XRD results (Fig. 6a). The *d*-values for CNC, CNC:TiO_2_, and GO/CNC:TiO_2_ were calculated to be 0.301, 0.363, and 0.442 nm, respectively. These values correspond to the (101) plane and increased slightly (0.295–0.442 nm), as seen in Fig. 6c and d.^53^ Furthermore, the change in d-spacing is ascribed to GO/CNC doping in the TiO_2_ lattice.

EDS analysis yielded information regarding the surface elemental composition of GO/CNC-doped TiO_2_, as shown in Fig. S2(a–d)†. According to the binding energies, the EDS spectrum contains peaks of carbon, titanium, oxygen, sulfur, and other elements. CNC contained 11.6 wt% elemental impurities of sulfur together with the main components of titanium (32.2%), carbon (30.1%), and oxygen (39.2% and 60.5%), as shown in Fig. S2(a and b)†. The oxygen peaks detected in the doped TiO_2_ confirm the incorporation of CNC and GO ions in the TiO_2_ lattice network. The elemental impurity can be ascribed to the dialysis of the CNC-containing sulfate groups, while the remainder resulted from the acid (H_2_SO_4_) hydrolysis of the cellulose nanocrystals.^54^ The tetragonal structure of TiO_2_ was revealed by XRD and its crystalline nature was established through SAED profiles. FTIR spectroscopy revealed the existence of functional groups and TiO_2_ (450 cm^−1^) in the fingerprint region. The presence of dopants (GO and C) and evaluation of the element constitution Ti and O were confirmed by EDS. The aforementioned results show the effective doping of GO and CNC in TiO_2_ with a QD morphology.

**Fig. 7:**
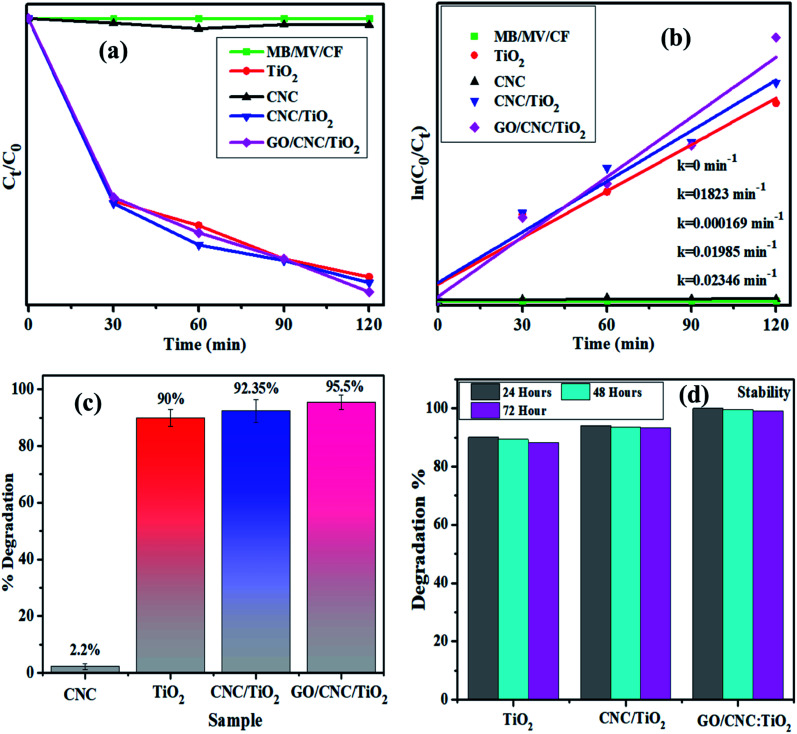
Plot of ratio (*C*_*t*_/*C*_0_) and −ln(*C*_*t*_/Co) *versus* time for dye reduction (a) and (b), respectively. (c) Degradation% plot and (d) stability of co-doped TiO_2_ samples.

The light-induced activity of the pristine and GO/CNC-doped TiO_2_ QDs is illustrated in Fig. 7. The absorbance presented as a function of time in Fig. 7a shows the enhanced MB/MV/CF removal over GO/CNC–TiO_2_. It was noticed that the GO/CNC-doped TiO_2_ achieved the highest MB/MV/CF reduction, *i.e.*, GO/CNC:TiO_2_, among the samples (Fig. 7c). The decrease in the recombination rate of excitons in GO/CNC:TiO_2_ is responsible for the improved photocatalytic activity of this system. The binary doping of GO and CNC may have induced defects in the lattice of TiO_2_ to act as trap sites for electrons, preventing them from reaching the holes. The PL spectra of the samples reflect this trend, with the pristine samples CNC and TiO_2_ having the maximum PL intensity, suggesting the highest recombination rate. PL peak intensity decreased for the binary-doped samples due to the slower exciton recombination in the lattice. The high photo-degradation efficiency may be due to the recombination of fewer excitons and increased visible light harvesting.

The Langmuir–Hinshelwood model can help explain how organic dyes degrade. The adsorption of dye molecules on the catalyst surface is enhanced by an in increase in the surface area of the nanocomposites, which have a higher number of active sites. The irradiation of nanocatalysts produces es^−^–h^+^ pairs, which assist in redox reactions with the surrounding dye molecules. The photo-generated electrons reduce the dye molecules and the holes oxidize them, transforming them into less hazardous compounds. The lowest degradation activity was shown by the pristine sample (90%, 68.2% and 71.42%), while the highest degradation of 95.5%, 74.23% and 78.52% was exhibited by the binary-doped sample at pH = 7 (neutral), pH = 12 (basic) and pH = 7 (neutral), respectively, as shown in Fig. 7c and S3.† Furthermore, for comparison, dye degradation in the dark was investigated and the corresponding results are shown in Fig. S4.† The Langmuir–Hinshelwood model was used to evaluate the reaction rate constant.^25^ The equation ln(*C*_*t*_/*C*_0_) = *k* was used to calculate the rate constants. The rate constants were attained from the slopes of the curves shown in Fig. 7b. For the undoped and doped samples, the calculated rate constants ranged from 0.1823 to 0.02346 min^−1^. Moreover, the dye concentration decreased continuously with an increase in the irradiation time.^53^ Experimental stability is a critical factor to be considered when evaluating a photocatalyst for wastewater treatment. The photocatalyst stability was examined by leaving the samples uninterrupted for three days and obtaining the absorption spectra from each sample every 24 h to record any variations in dye degradation. The degradation of the dye was monitored spectrophotometrically every 24 h and the corresponding results are depicted in Fig. 7d. The GO/CNC-doped TiO_2_ photocatalyst demonstrated outstanding stability and proved to be a promising photocatalyst.

The *in vitro* antimicrobial strength of the GO/CNC-doped TiO_2_ against *E. coli* and *S. aureus* retrieved from caprine mastitis fluid was evaluated. The findings show the superior additive antibacterial effect of GO/CNC-doped TiO_2_ against *E. coli* compared to *S. aureus*, as shown in Fig. S5.† The statistically important inhibitory areas (*p* < 0.05) were measured at a minimum and maximum dosage of (0.95 ± 0.05–1.03 ± 0.12 mm) and (1.67 ± 0.06–2.10 ± 0.05 mm) against *S. aureus*, while (3.36 ± 0.07–3.49 ± 0.05 mm) and (4.49 ± 0.05–5.21 ± 0.02 mm) against *E. coli*, respectively. TiO_2_ as a control specimen demonstrated a value of zero against *E. coli* at the minimum and maximum dosage. Ciprofloxacin decreased *S. aureus* and *E. coli* growth by (7.75 ± 0.00 mm) and (7.15 ± 0.00 mm), respectively, in contrast to DIW (0 ± 0.00 mm), as shown in Table S1.† The cumulative bactericidal potency of the GO/CNC-doped TiO_2_ material towards Gram-negative relative to Gram-positive was significant (*p* < 0.05). The oxidative damage generated by QDs depends on their form, concentration, and size, which are inversely proportional to the properties of the doped substances.^55,56^ The reactive oxygen species (ROS) released from QDs remain efficiently in the pathogen cell membrane, resulting in cytoplasmic extrusion and pathogen death.^57^ Secondly, the heavy cationic association of Ti^4+^ at higher concentrations with the negative pathogenic cell leads to cell deterioration and bacteria death.^26^

**Fig. 8:**
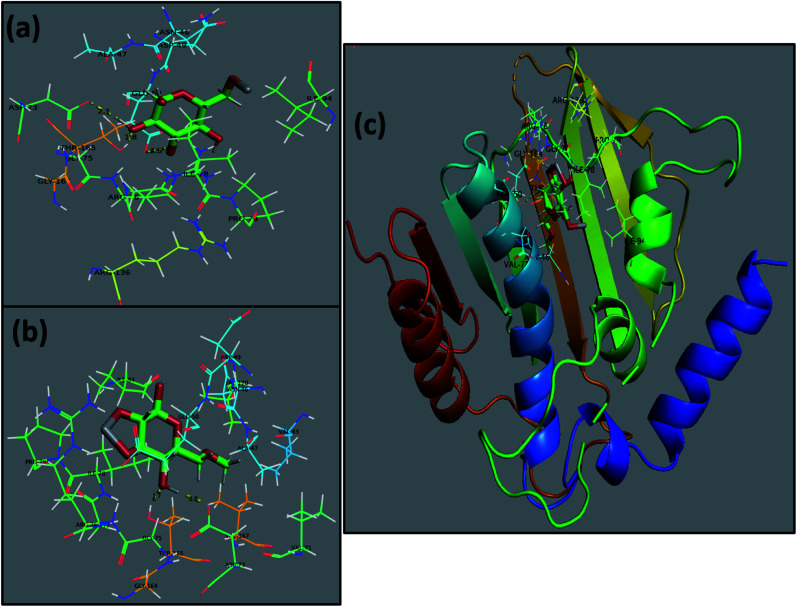
Binding interaction pattern of DNA gyrase from *E. coli* (a), TiO_2_–CNC (b), and GO/CNC-doped TiO_2_ (c) and superimposed docked complexes of TiO_2_–CNC and GO/CNC-doped TiO_2_ with DNA gyrase.

The use of computational techniques for mechanistic studies to rationalize the mystery behind biological activities and functions is well reported.^26,34,58^ Enzymes are considered to play the main role in bacterial infection, and thus inhibiting their activity serves to combat the caused infection.^58,59^ Here, the molecular docking predictions of TiO_2_–CNC and GO/CNC-doped TiO_2_ NPs against the FabI and DNA gyrase enzyme of *E. coli* and *S. aureus* revealed that they are the potent inhibitors of the given enzyme targets, as suggested by their binding tendency inside their binding pockets, respectively. The docked complex obtained for TiO_2_–CNC against FabI of *E. coli* revealed H-bonding with Ala21 (2.6 Å), Val14 (2.0 Å), and Ser19 (2.7 Å) having a binding score −9.117 kcal mol^−1^. The GO/CNC-doped TiO_2_ also showed a good binding score involving H-bond with Lys163 (2.4 Å and 2.5 Å) and Thr194 (2.5 Å) together with C–C interaction with Ser91 and Gly13, as depicted in Fig. S6.† The docked complex (−8.763 kcal mol^−1^) obtained for TiO_2_–CNC against FabI of *S. aureus* revealed the involvement of three amino acids, *i.e.*, Ser44 (2.3 Å), Arg40 (2.4 Å and 2.8 Å) and Lys41 (2.4 Å), interacting through H-bonds, while GO/CNC-doped TiO_2_ revealed H-bonding with Ala95 (2.2 Å and 2.4 Å) and metal-contact with Ser197 (binding score of −5.974 kcal mol^−1^), as shown in Fig. S7.† Furthermore, the docking predictions for DNA gyrase from *E. coli* were also assessed to analyze the binding potential of TiO_2_–CNC (binding score: −6.849 kcal mol^−1^) and GO/CNC-doped TiO_2_ (binding score: −4.796 kcal mol^−1^) against active sites. The major interactions observed for TiO_2_–CNC were H-bonding with Asp73 (2.1 Å), Thr165 (1.8 Å), and Glu50 (2.5 Å), while GO/CNC-doped TiO_2_ interacted through H-bonding with Asp73 (2.0 Å) and Thr165 (1.7 Å), as depicted in Fig. 8.

## 4. Conclusion

This study utilized a cost-effective chemical precipitation approach to produce novel GO/CNC-doped TiO_2_ QDs with strong photocatalytic and antimicrobial capabilities. The influence of GO/CNC doping on the phase structure, morphology, elemental analysis, and optical properties of TiO_2_ was investigated. The TiO_2_ QDs were found to have a tetragonal structure with an anatase phase, and an increase in crystallinity was observed using CNC/GO. The crystallite size increased from 4.36 to 6.28 nm upon doping with GO/CNC. The FTIR peaks between 450 and 1000 cm^−1^ are ascribed to the Ti–O–Ti vibrations, confirming the presence of TiO_2_. The UV-Vis spectra revealed a redshift in the absorption window upon doping of GO/CNC, and resulting in a decrease in Eg from 3.22 to 2.96 eV. The FESEM and HRTEM images revealed a significant degree of QD aggregation, and a *d*-spacing of ∼0.301 nm was determined for the sample containing the maximum doping concentration. Furthermore, the GO/CNC-doped TiO_2_ samples exhibited efficient degradation (10.46%) of MB/MV/CF dye with a rate constant of 0.0015 min^−1^. By comparing *E. coli* and *S. aureus* isolates of caprine mastitis, GO/CNC-doped TiO_2_ exhibited enhanced bactericidal action and synergism against Gram-negative bacteria. DNA gyrase and FabI inhibition were proposed as the probable mechanism for the bactericidal action of TiO_2_–CNC and GO/CNC-doped TiO_2_, as revealed by *in silico* molecular docking investigation. The fabricated QDs developed through an innovative and cost-effective methodology can be potential photocatalysts and bactericidal agents against infectious bacterial etiologies as future medicinal materials.

## Conflicts of interest

A conflict of interest has not been identified by the authors.

## Supplementary Material

NA-004-D2NA00383J-s001
