# Supplementary material for: Photocatalytic and antibacterial activity of graphene oxide/cellulose-doped TiO2 quantum dots: in silico molecular docking studies
Source: Nanoscale Adv. 2022 Jul 27;4(18):3764–76. doi: 10.1039/d2na00383j (PMC9470022; doi:10.1039/d2na00383j)
Supplement: NA-004-D2NA00383J-s001 [file NA-004-D2NA00383J-s001.pdf]

Electronic Supplementary Material (ESI) for Nanoscale Advances.
This journal is © The Royal Society of Chemistry 2022

**Supplementary Material**

# Photocatalytic and Antibacterial activity of graphene oxide/cellulose-doped $TiO_2$ quantum dots: In silico molecular docking studies

Muhammad Ikram[a]*, Fahad Rasheed[b], Ali Haider[c], Sadia Naz[d], Anwar Ul-Hamid[e]*, Anum Shahzadi[e], Junaid Haider[f], Iram Shahzadi[g]; Shaukat Hayat[b], Salamat Ali[b]

[a]Solar Cell Application Research Lab, Department of Physics, Government College University Lahore, Lahore, 54000, Punjab, Pakistan

[b]Department of Physics, Riphah Institute of Computing and Applied Sciences (RICAS), Riphah International University, 14 Ali Road, Lahore, Pakistan

[c]Department of Clinical Sciences, Faculty of Veterinary and Animal Sciences, Muhammad Nawaz Shareef University of Agriculture, Multan, 66000, Pakistan

[d]Tianjin Institute of Industrial Biotechnology, Chinese Academy of Sciences, Tianjin 300308, China.

[e]Core Research Facilities, King Fahd University of Petroleum & Minerals, Dhahran, 31261, Saudi Arabia

[f]Faculty of Pharmacy, The University of Lahore, Lahore, Pakistan

[g]Punjab University College of Pharmacy, University of the Punjab, 54000, Pakistan

*Corresponding authors emails: [a]dr.muhammadikram@gcu.edu.pk, [e]anwar@kfupm.edu.sa

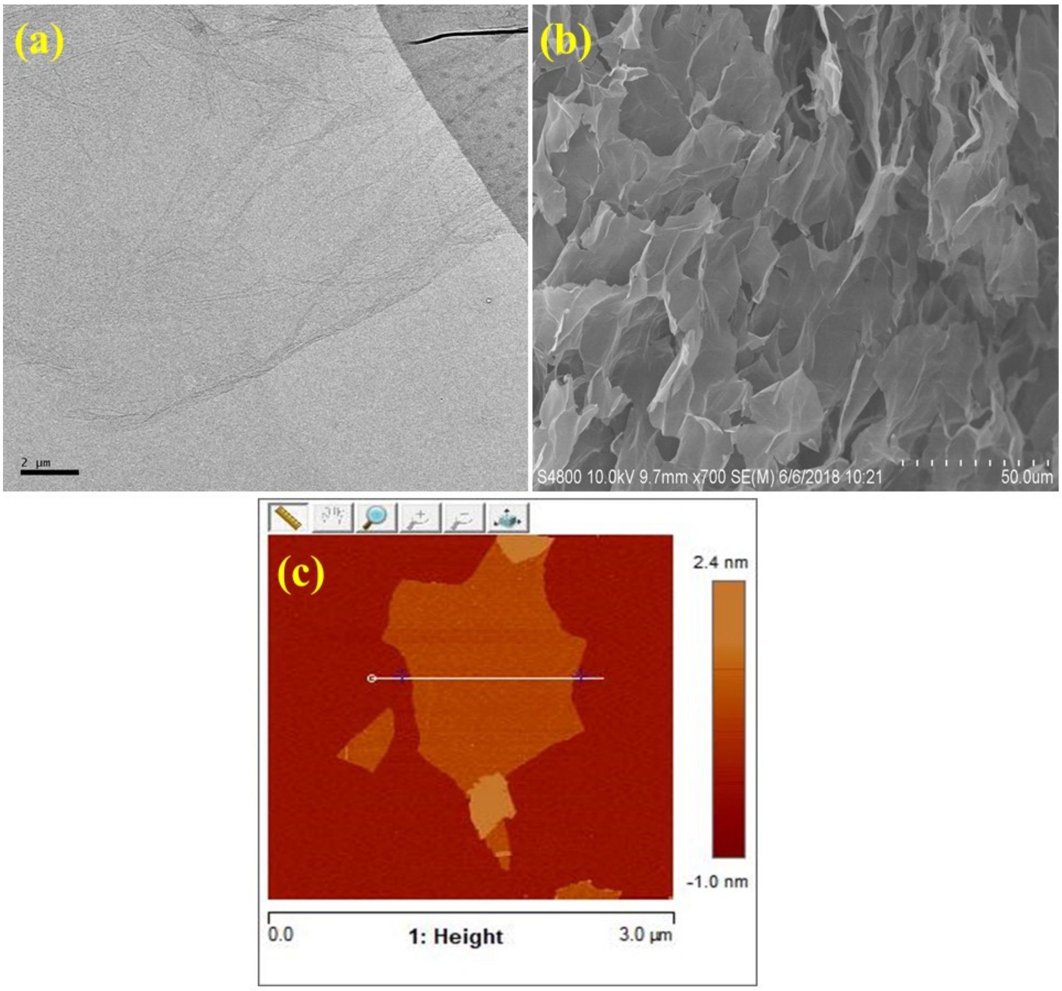


**Fig. S1:** (a) HR-TEM, (b) SEM, and (c) Atomic Force Microscopy images of GO

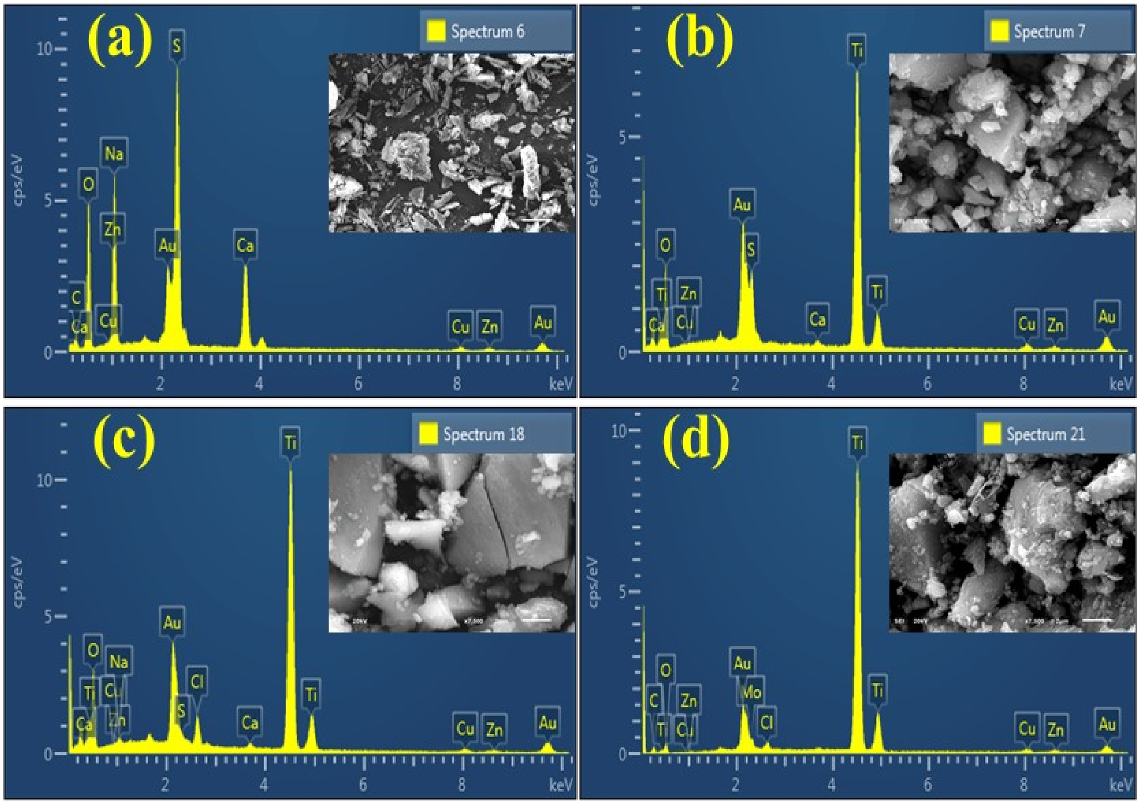


**Fig. S2:** Energy dispersive x-ray spectroscopy (EDS) results obtained from (a) CNC, (b) $TiO_2$, (c) CNC:$TiO_2$ and (d) GO:CNC:$TiO_2$

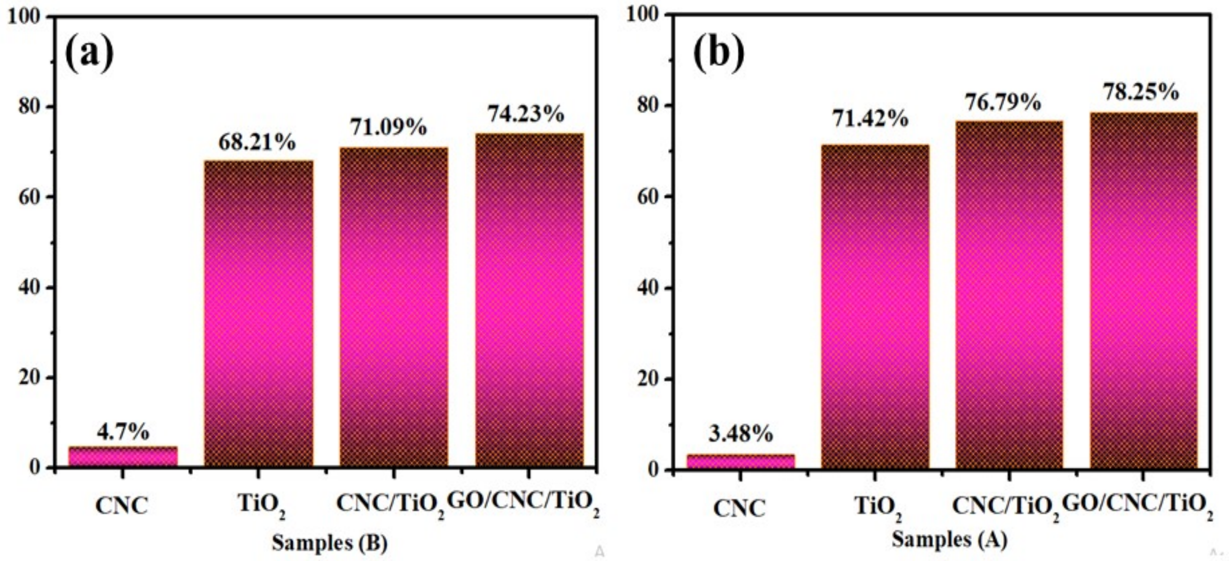


**Figure S3.** Photocatalysis of CNC, $TiO_2$, CNC/$TiO_2$, GO/CNC/$TiO_2$ in (a) basic and (b) acidic medium, respectively

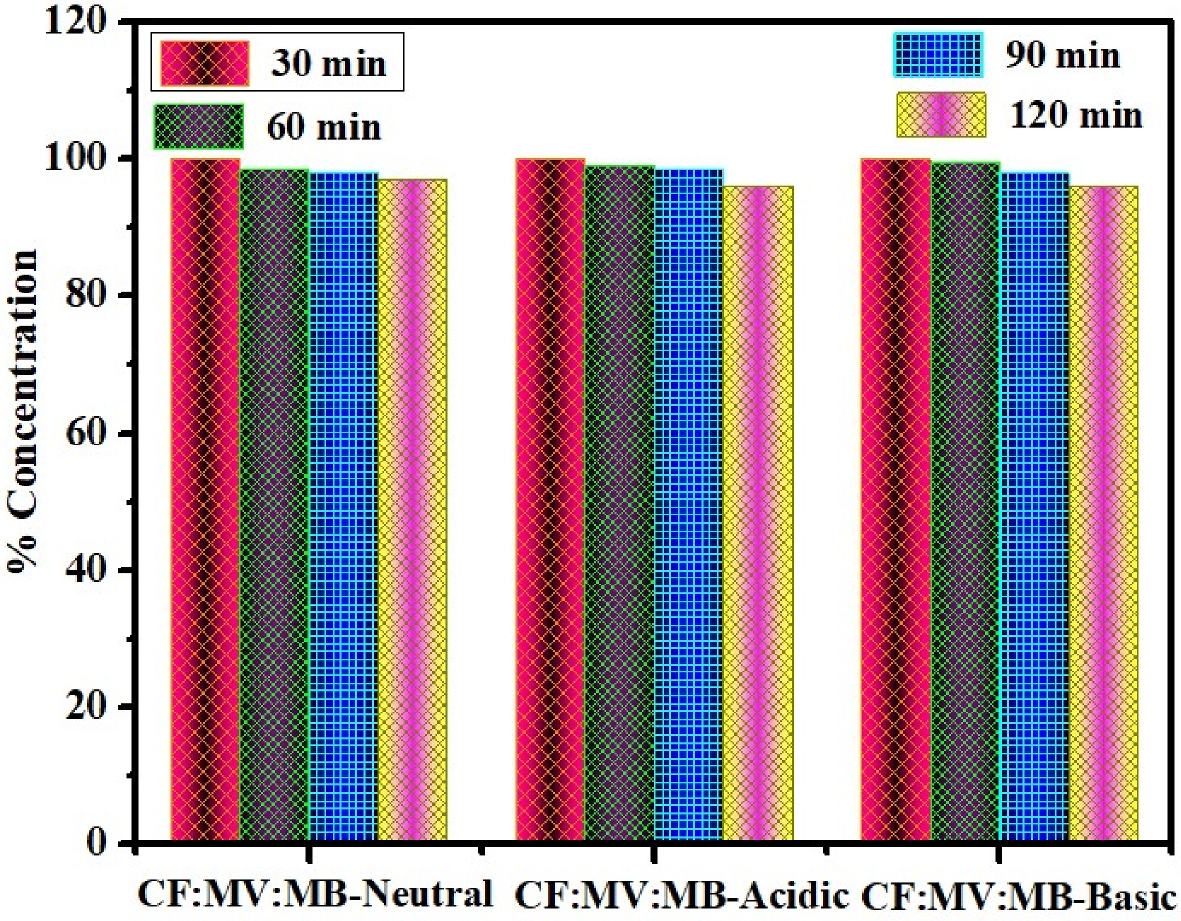


**Figure S4.** Dye degradation in the dark for comparison.

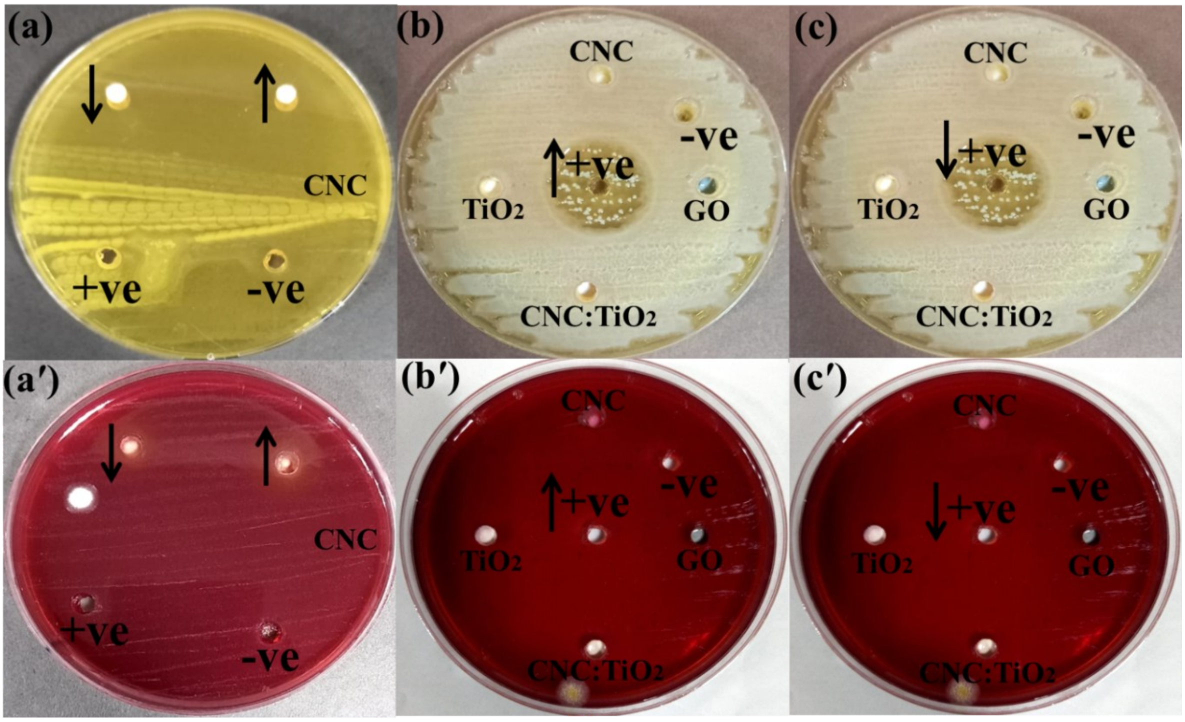


Fig. S5: In vitro antibacterial activity of (a, a′) CNC and (b, b′-c, c′) bare and doped $TiO_2$ for low and high concentration against SA and EC, respectively.

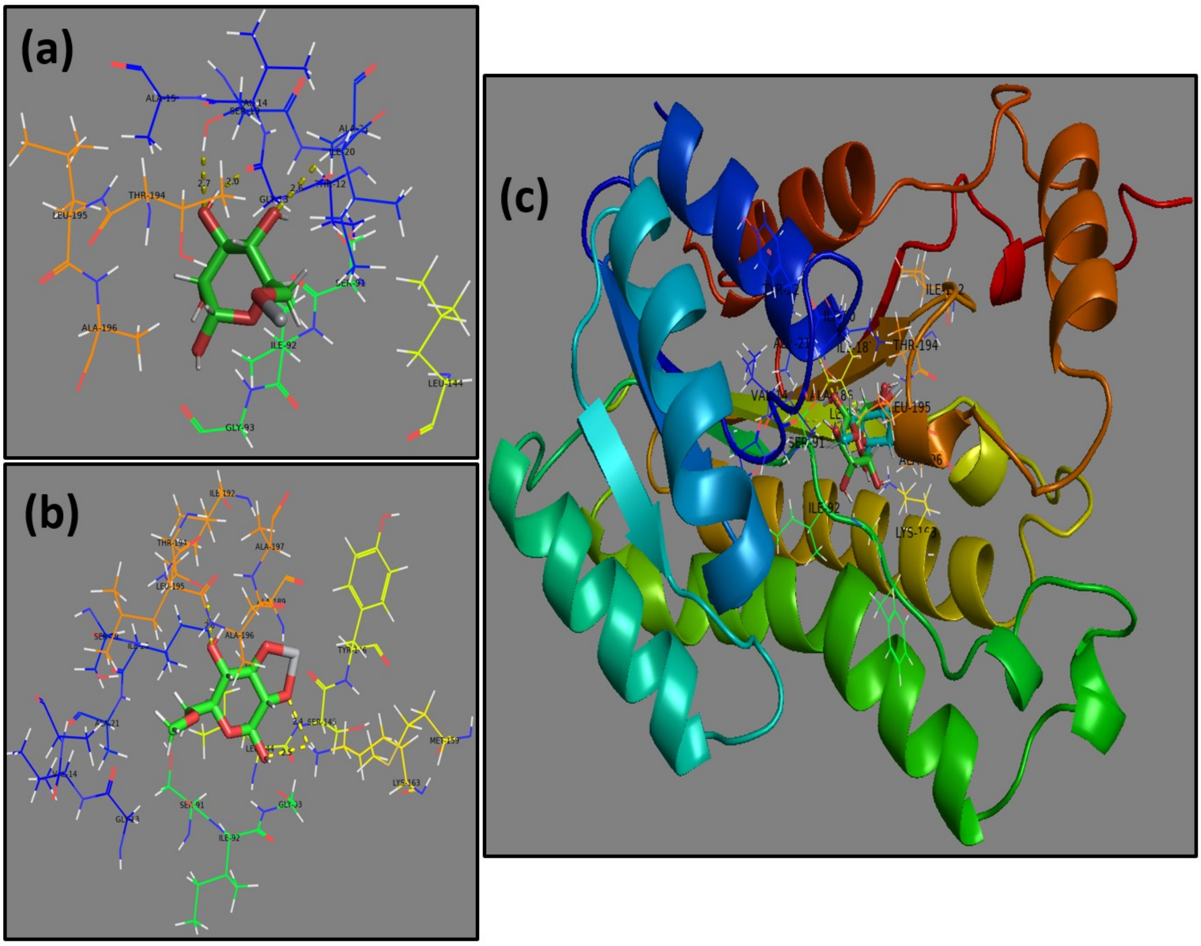


**Figure S6:** Binding interaction pattern inside active pocket of FabI from *E. coli* (a). $TiO_2$-CNC, (b). GO/CNC-doped $TiO_2$, (c). Superimposed docked complexes of $TiO_2$-CNC and GO/CNC-doped $TiO_2$ with FabI

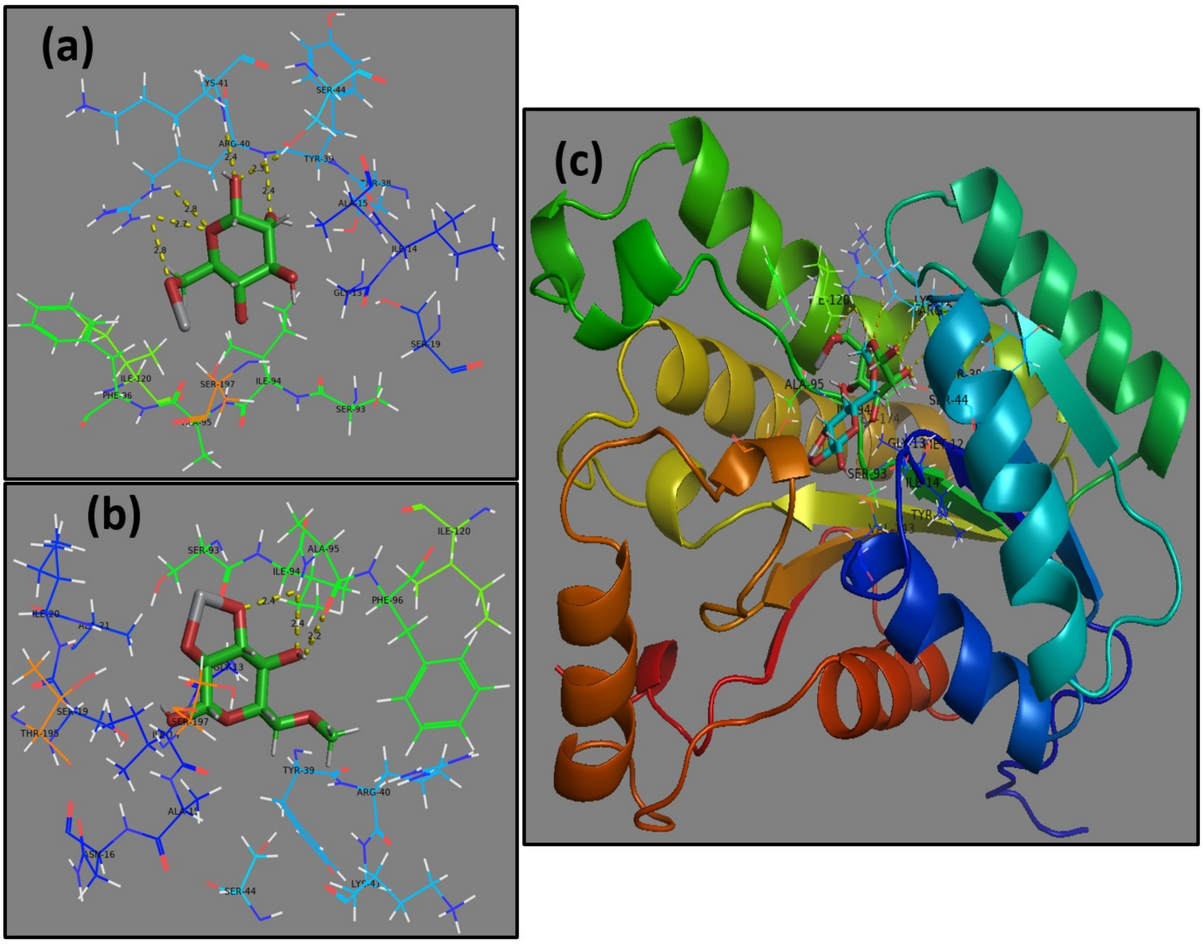


**Figure S7:** Binding interaction pattern inside active pocket of FabI from *S. aureus* **(a).** $TiO_2$-CNC, **(b).** GO/CNC-doped $TiO_2$, **(c).** Superimposed docked complexes of $TiO_2$-CNC and GO/CNC-doped $TiO_2$ with FabI

Figure S8 demonstrated that the MB degradation occurs around λ of 665 nm for MV = 605 and CF = 290 nm, respectively. Dyes degrade gradually under illumination upon doping GO/CNC doped TiO2, while observed UV-Vis spectra are given below.

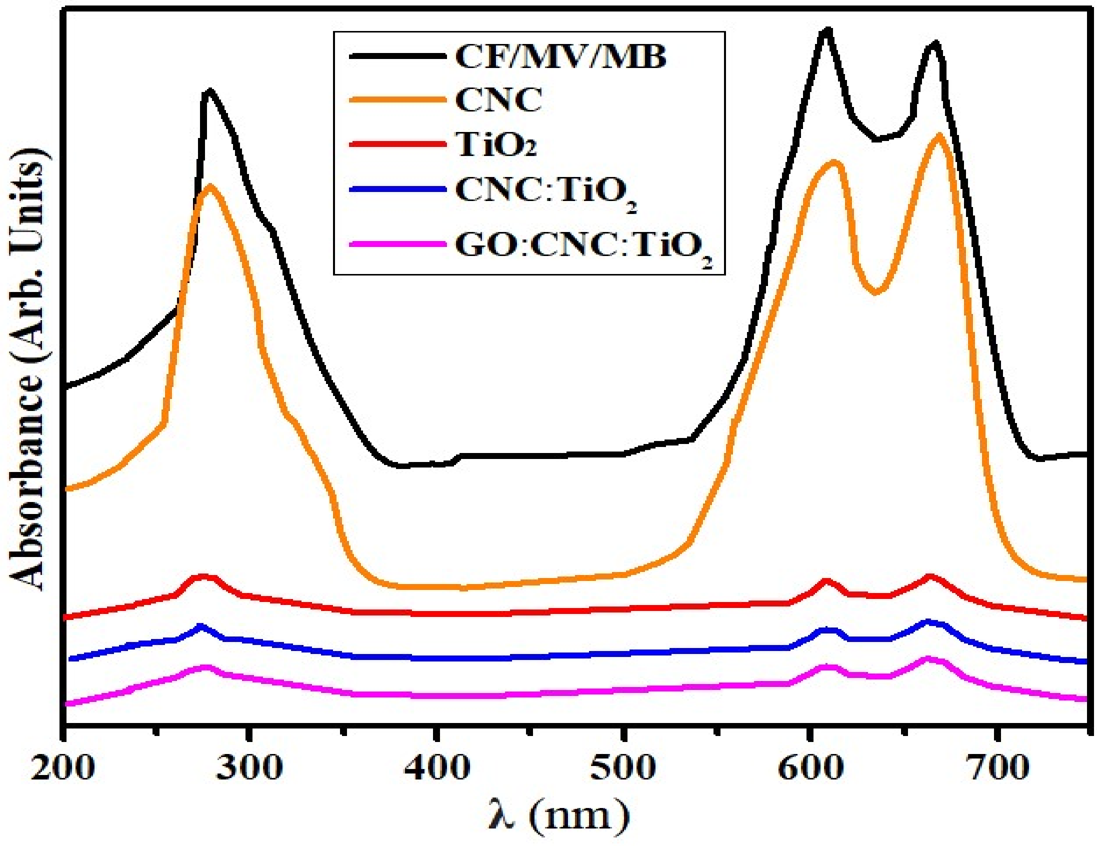


**Figure S8**. Represents the CF, MV, and MB absorption peaks for the neutral medium of the prepared sample

Table S1 Bactericidal action of GO/CNC:$TiO_2$

| Samples | *S. aureus* Inhibition zone[a] (mm) | | *E. coli* Inhibition zone[b] (mm) | |
|---|---|---|---|---|
| | 1 % | 2 % | 1 % | 2 % |
| CNC | 0.95±0.05 | 1.67±0.06 | 3.36±0.07 | 4.49±0.05 |
| $TiO_2$ | 0±0.00 | 1.26±0.03 | 0±0.00 | 0±0.00 |
| CNC:$TiO_2$ | 0±0.00 | 1.73±0.07 | 3.17±0.02 | 4.52±0.02 |
| GO/CNC:$TiO_2$ | 1.03±0.12 | 2.10±0.05 | 3.49±0.05 | 5.21±0.02 |
| Ciprofloxacin | 7.75±0.00 | 7.75±0.00 | 7.15±0.00 | 7.15±0.00 |
| DIW | 0±0.00 | 0±0.00 | 0±0.00 | 0±0.00 |

[a] Inhibition zone (mm) of GO/CNC-doped $TiO_2$ for *S. aureus*

[b] Inhibition zone measurements for *E. coli.*
